# Pain Perception in Affective Disorders: The Role of Chronotype, Biological Rhythm, and Sleep Quality-Preliminary Findings

**DOI:** 10.1192/j.eurpsy.2026.10933

**Published:** 2026-07-31

**Authors:** F. B. Parlakkaya Yıldız, E. Çetintaş, Z. Çelik, A. Kurtulmuş Çalış, S. N. Keskin

**Affiliations:** Psychiatry, Istanbul Medeniyet University, Göztepe Prof Dr Süleyman Yalçın City Hospital, ISTANBUL, Türkiye

## Abstract

**Introduction:**

Pain perception is frequently altered in affective disorders, with both unipolar and bipolar depression linked to reduced pain thresholds and tolerance (Bair et al., 2003). Biological rhythm disturbances and poor sleep quality are also closely related to the onset and course of depression. Circadian misalignment has been shown to increase pain sensitivity and vulnerability to chronic pain (Golombek & Rosenstein, 2010), while impaired sleep further exacerbates symptoms (Finan et al., 2013). However, the combined role of chronotype, biological rhythms, and sleep quality in pain perception remains underexplored.

**Objectives:**

This study aimed to investigate the influence of chronotype, biological rhythm disturbances, and sleep quality on pain perception in affective disorders. Specifically, we compared pain threshold and tolerance in individuals with unipolar depression, bipolar depression, and healthy controls, and examined whether chronotype (MEQ), biological rhythms (BRIAN), and sleep quality (PSQI) predict pain sensitivity beyond depressive symptom severity.

**Methods:**

Ninety participants were recruited: 30 with unipolar depression, 30 with bipolar depression (depressive episode), and 30 healthy controls, from the psychiatry outpatient clinic of Göztepe City Hospital. Diagnoses were established according to DSM-5. Assessments included sociodemographic data, depressive severity (BDI), chronotype (MEQ), biological rhythms (BRIAN), and sleep quality (PSQI). Pain threshold and tolerance were measured on the nondominant forearm using a pressure algometer (Chesterton et al., 2007). Data were analyzed using descriptive statistics, chi-square tests, ANOVA or Kruskal–Wallis with post-hoc comparisons, independent t-tests, Pearson/Spearman correlations, and multiple regression, with significance set at p<0.05.

**Results:**

Groups were broadly comparable in demographic characteristics. Both unipolar and bipolar depression showed poorer sleep quality (PSQI, p<0.001) and greater biological rhythm disturbance (BRIAN total, p<0.001) than controls.P ain tolerance was lower in patients (controls > bipolar > unipolar; p=0.003), whereas pain threshold did not differ (p=0.323). In unipolar depression, higher depressive severity correlated with lower pain threshold, and regression analyses indicated that depression severity -but not sleep or rhythm indices -predicted pain perception. Data collection and analyses are ongoing to further validate and extend these preliminary findings.

**Conclusions:**

Patients with affective disorders showed poorer sleep, greater rhythm disruption, and lower pain tolerance than controls. Reduced pain threshold was linked mainly to depression severity, while sleep and circadian disturbances, though related to symptom burden, did not independently predict pain perception.These findings underscore the importance of integrating pain assessment into affective disorder management.

**Disclosure of Interest:**

None Declared

